# A Novel and Robust Prognostic Model for Hepatocellular Carcinoma Based on Enhancer RNAs-Regulated Genes

**DOI:** 10.3389/fonc.2022.849242

**Published:** 2022-05-12

**Authors:** Wei Zhang, Kegong Chen, Wei Tian, Qi Zhang, Lin Sun, Yupeng Wang, Meina Liu, Qiuju Zhang

**Affiliations:** ^1^ Department of Biostatistics, School of Public Health, Harbin Medical University, Harbin, China; ^2^ Department of Cardio-Thoracic Surgery, The Second Affiliated Hospital of Harbin Medical University, Harbin, China; ^3^ Department of Ultrasound, The First Affiliated Hospital of USTC, Division of Life Sciences and Medicine, University of Science and Technology of China, Hefei, China

**Keywords:** hepatocellular carcinoma, enhancer RNAs, signature, prognosis, AKR1C3

## Abstract

Evidence has demonstrated that enhancer RNAs (eRNAs) play a vital role in the progression and prognosis of cancers, but few studies have focused on the prognostic ability of eRNA-regulated genes (eRGs) for hepatocellular carcinoma (HCC). Using gene expression profiles of HCC patients from the TCGA-LIHC and eRNA expression profiles from the enhancer RNA in cancers (eRic) data portal, we developed a novel and robust prognostic signature composed of 10 eRGs based on Lasso-penalized Cox regression analysis. According to the signature, HCC patients were stratified into high- and low-risk groups, which have been shown to have significant differences in tumor immune microenvironment, immune checkpoints, HLA-related genes, DNA damage repair-related genes, Gene-set variation analysis (GSVA), and the lower half-maximal inhibitory concentration (IC50) of Sorafenib. The prognostic nomogram combining the signature, age, and TNM stage had good predictive ability in the training set (TCGA-LIHC) with the concordance index (C-index) of 0.73 and the AUCs for 1-, 3-, and 5-year OS of 0.82, 0.77, 0.74, respectively. In external validation set (GSE14520), the nomogram also performed well with the C-index of 0.71 and the AUCs for 1-, 3-, and 5-year OS of 0.74, 0.77, 0.74, respectively. In addition, an important eRG (AKR1C3) was validated using two HCC cell lines (Huh7 and MHCC-LM3) *in vitro*, and the results demonstrated the overexpression of AKR1C3 is related to cell proliferation, migration, and invasion in HCC. Altogether, our eRGs signature and nomogram can predict prognosis accurately and conveniently, facilitate individualized treatment, and improve prognosis for HCC patients.

## 1 Introduction

Hepatocellular carcinoma (HCC) is the most common type (approximately 90%) of primary liver cancer, which has already become the sixth most commonly diagnosed cancer (906,000 new cases) and the third leading cause of cancer death (830,000 deaths) worldwide in 2020 (1). Chronic infection of hepatitis B virus (HBV) or hepatitis C virus (HCV), cirrhosis, excessive alcohol consumption, and type 2 diabetes are the main risk factors for HCC (2). HCC has a very poor prognosis due to its advanced stage, rapid progression, high recurrence rate, and limited treatment options (3). Traditionally, tumor stage is a widely used basis for predicting the prognosis of patients with HCC (4, 5). However, prognosis in HCC is complex and highly heterogeneous (6, 7), patients in the same tumor stage may present significantly different prognosis. A valuable, accurate strategy is still an urgent need to predict HCC prognosis.

Previous research has proposed that serum biochemical biomarkers, such as hepatic growth factor, osteopontin, BALAD scoring model composed of alpha-fetoprotein (AFP), alpha-fetoprotein lens culinaris agglutin-3 (AFP-L3), and Des-γ-carboxy prothrombin (DCP) could be used for predicting prognosis (8–10). With the development of high-throughput sequencing technology, genes were considered to be important factors in predicting survival of patients with HCC (11–14). Furthermore, gene signatures based on multigene expression, and nomograms including gene signature and clinical information have been constructed for patients’ prognosis in many published studies (15–17). However, it is still necessary to further mine omics data in combination with clinical characteristics to discover a novel and reliable prognostic model for HCC and guide patients’ individualized treatment.

Enhancer RNAs (eRNAs) are non-coding RNAs transcribed by enhancers that mediate the activation of target genes (18, 19). In human cancers, eRNAs can contribute to the activation of oncogenes or oncogenic signaling pathways and can be induced by oncogenes or tumor suppressors to directly participate in tumor promotion or inhibition process (20–22). Furthermore, eRNAs can also bind to DNA, proteins (e.g., transcription factors, cofactors, RNA-binding proteins) to regulate their function and activity (23). Super-enhancers, the clusters of enhancers, also play prominent roles in dysregulation of oncogenes expression, tumor suppressor genes expression, process of tumorigenesis, and proliferation of cancer cells (23–25). Although some data portals have already annotated a mass of enhancers, the direct interaction between eRNA and its target genes has not been elaborated until researchers integrated data from TCGA and other projects (26–28) and developed eRic data portal including a global eRNA-gene regulatory network across 31 cancer types in 2019 (29). This portal facilitates a deeper investigation of relationships between eRNAs and cancers for biomedical researchers. However, few studies have focused on eRNA-regulated genes (eRGs) to construct prognostic models of HCC patients for the management of individualized treatment.

This study attempted to construct a novel prognostic gene signature composed of eRGs and nomogram combining the signature with clinical characteristics by using univariate and Lasso-penalized Cox regression analyses in TCGA-LIHC dataset and validate it in GSE14520 dataset. We demonstrated the application potential of the signature and nomogram through bioinformatics methods. In addition, we validated the effect of important eRG in the signature on cell proliferation, migration, and invasion of HCC using two HCC cell lines (Huh7 and MHCC-LM3) *in vitro*.

## 2 Materials and Methods

### 2.1 Data Collection and Processing

Data used in this study were all publicly available. mRNA expression and clinical data of 374 LIHC patients were obtained from the Cancer Genome Atlas (TCGA) data portal (https://portal.gdc.cancer.gov/), and the eRNA expression profile across these TCGA samples as well as eRNA target genes were acquired from the enhancer RNA in cancers (eRic) data portal (https://hanlab.uth.edu/eRic/). The raw mRNA data in TCGA was processed to fragments per kilobase of transcript per million mapped reads (FPKM) and transformed based on log2 (log2FPKM). Patients with insufficient survival information or follow-up period less than 30 days were excluded, and 324 HCC samples were selected as the training set for subsequent analysis. Microarray dataset GSE14520 -GPL3921 includes 219 HCC patients with integral clinical information and survival time longer than 30 days were downloaded from the Gene Expression Omnibus (GEO) database (https://www.ncbi.nlm.nih.gov/geo/) as the external validation set.

### 2.2 Construction and Validation of eRGs Signature

In this section, we identified eRNAs related to survival in HCC patients and the target genes regulated by them. Based on the target genes, an eRGs signature was constructed and validated for predicting prognosis.

Kaplan-Meier analysis and univariate Cox regression were used to screen eRNAs related to survival in TCGA dataset, and only the eRNAs with *p* < 0.05 in both above analyses were selected. Then the target genes of these eRNAs were identified by referring to the eRic database.

The target genes of survival-related eRNAs in TCGA-LIHC were firstly subjected to univariate Cox regression analysis and genes with *p* < 0.05 were considered as the candidate prognostic eRGs. Subsequently, Lasso-penalized Cox regression analysis was performed to build the prognostic eRGs signature, and 10-fold cross-validation was carried out to determine the optimal penalty parameter using “glmnet” package in R. Then the prognostic eRGs signature was established, and the risk score of each patient can be calculated based on the corresponding coefficients of genes from the Lasso Cox regression model (β) and their expression level:


$$riskScore=\sum_{i=1}^{m}\beta_{i}\times Exp_{i}$$

where m is the number of the genes in signature, *β_i_* is the coefficient from Lasso Cox analysis, *Exp_i_* is the expression level of the eRGs in signature. The median risk score was used as the cutoff value to stratify the HCC patients into high risk and low risk groups. Thereafter, we performed a log-rank test to compare the survival rates between the two groups and plotted Kaplan-Meier survival curves using “survival” and “survminer” package in R. Time-dependent receiver operating characteristic (ROC) curves for 1-, 3-, and 5-year OS were also drawn based on the risk score using “timeROC” package in R to assess the prognostic performance of the signature. To validate the predictive capability and generalization of the eRGs signature, HCC patients in GSE14520 dataset with intact survival and clinical information were considered as the external validation set. Risk scores of these patients were calculated with the same model of prognostic eRGs signature, and Kaplan-Meier survival curves analysis and the ROC curves analysis were also conducted in this dataset.

### 2.3 Construction and Validation of Prognostic Nomogram

We performed univariate Cox regression and multivariable Cox regression analysis on the risk score of the gene signature and other clinical features (including age, TNM stage, AFP level, and BMI) in TCGA dataset to identify the independent prognostic factors, and factors with *p* < 0.05 were deemed statistically significant. The proportional hazard assumption of the model was tested by Schoenfeld residuals test. Then a nomogram based on these independent prognostic factors was developed using “rms” package in R to predict the overall survival time of HCC patients. Nomogram is a graphical representation of a complex mathematical formula (30). In medicine, a nomogram is usually used to graphically describe a statistical prognostic model that generates the probability of a particular individual’s clinical events (such as cancer recurrence or overall survival), which can be conductive to personalized medicine (31). Calibration curves were utilized to investigate the consistency between the nomogram-predicted probabilities and the actual survival rates, and decision curves analysis (DCA) was conducted to assess the clinical predictive value of the nomogram. Furthermore, time-ROC curves were also plotted in TCGA dataset to evaluate the prognostic performance of the nomogram. Based on the nomogram in the training set, calibration curves analysis, DCA analysis and time-ROC curves analysis were all performed in the external validation set GSE14520 to further validate the results.

### 2.4 Enrichment Analyses and Gene-Set Variation Analysis

Gene Ontology (GO) and Kyoto Encyclopedia of Genes and Genomes (KEGG) enrichment analyses were performed with survival-related eRGs set, and terms with adjusted *p* < 0.05 were considered as significant enrichment. Gene Set Enrichment Analysis (GSEA) was also conducted on the genes in the survival-related signature to explore the potential pathways by using “clusterProfiler” package in R (32). After that, we applied gene-set variation analysis (GSVA) using “GSVA” package and “limma” package in R to identify the different pathways between the two different risk groups following the criteria of |log2FC| > 0.2 and *p* < 0.05 (33). The annotated gene set used in GSEA and GSVA was “c5.all.v6.2.symbols.gmt”, which can be downloaded from the Molecular Signatures Database (MSigDB).

### 2.5 Exploration of Immunotherapy Effect and Immune Landscape

Immune checkpoint inhibitors play an important role in the treatment of liver cancer (34), so we compared the differences in the expressions of some common immune checkpoints (CTLA4, PD-L1, TIGIT, and HAVCR2) between high risk patients and low risk patients using Wilcoxon rank-sum test. We also calculated the proportion of 22 immune-infiltrating cells in each patient using “Cell type Identification By Estimating Relative Subsets Of RNA Transcripts (CIBERSORT)” algorithm (35), and the patients with *p* < 0.05 were used for difference comparison in the two sub-groups. In addition, we utilized TIMER (Tumor Immune Estimation Resource) database to further explore the correlation between risk score and six immune cells including B cells, CD4+ T cells, CD8+ T cells, macrophages, neutrophils, and dendritic cells (36). Human leukocyte antigen (HLA) is the major histocompatibility complex (MHC) in human, which goes together with the function of human immune system. Therefore, we also validated the differences in HLA-related genes between the two groups.

### 2.6 Evaluation of Drug Sensitivity

Half maximal inhibitory concentration (IC50) is a widely used indicator to evaluate the sensitivity of drug therapy (37). Sorafenib, a targeted therapy drug, has been considered the standard treatment for patients with hepatocellular carcinoma (HCC) since 2007 (38). Therefore, we estimated the IC50 of Sorafenib in high- and low-risk patients and determined whether there is a difference in the sensitivity of different patients to sorafenib. The drug IC50 was estimated using “pRRophetic” package in R based on Genomics of Drug Sensitivity in Cancer (GDSC, https://www.cancerrxgene.org/) cell line expression data and TCGA-LIHC gene expression data (39, 40).

### 2.7 Identification of Transcription Factors Related to the Genes in Signature

Transcription factor (TF) list was downloaded from Cistrome (http://cistrome.org/), and the expression level of these TF were extracted from 324 TCGA-LIHC patients. Then we performed Spearman correlation test between the expression of eRGs in the signature and these TFs. The gene-TF pairs with absolute Spearman correlation coefficients > 0.4 and *p* < 0.05 were selected to further discussion.

### 2.8 *In Vitro* Experimental Validation of AKR1C3 in HCC Cell Lines

#### 2.8.1 Cell Culture, Transfection and Sorafenib Treatment

Human hepatocellular carcinoma cell lines Huh7 and MHCC-LM3 were obtained from the Cell Bank of the Chinese Academy of Sciences (Beijing, China), both types of cells were cultured in DMEM-6429 (Sigma, MO, USA) with 10% fetal bovine serum (FBS, HyClone, Logan, UT, USA). The siRNA sequences targeting AKR1C3 used in this study were as follows: si-AKR1C3-1:(5’-CCAAACACCAGUGUGUAAATT-3’, 5’-UUUACACACUGGUGUUUGGTT-3’); and si-AKR1C3-2:(5’-GGAACUUUCACCAACAGAUTT-3’, 5’-AUCUGUUGGUGAAAGUUCCTT-3’) and negative controls (si-AKR1C3-NC); AKR1C3 overexpression plasmid:(5’-ATGGATTCCAAACACCAGTGT-3’, 5’-TTAATATTCATCTGAATATGG-3’) and empty plasmids were regarded as negative controls (NC) purchased from the Nantong Biomics Biotechnologies company. Transfection was performed using Lipofectamine 3000 (Thermo Fisher Scientific, Inc) according to the manufacturers’ instructions. The cells were harvested 48 hours after transfection. In addition, Sorafenib (8μM) was added to Huh7 and MHCC-LM3 cells prior the incubation.

#### 2.8.2 Protein Extraction and Western Blot Analysis

Total protein was extracted using RIPA buffer and quantified with a BCA kit (Beyotime Biotechnology). Protein separation was performed using 10% SDS-PAGE and then transferred to PVDF membranes (Merck Millipore). Following being blocked with 5% non-fat milk for 2 h at 25°C, the PVDF membranes were incubated with AKR1C3(Abcam; 1:1000; ab209899) and GAPDH (Abcam; 1:5000; ab9485), and then incubated with a secondary antibody (Cell Signaling Technology) for 1 h. PVDF membranes were scanned by a chemiluminescence system.

#### 2.8.3 Cell Viability and Colony Formation Assays

EdU cell proliferation assay was performed using a commercial EdU Kit (UE, China) according to the manufacturer’s protocol. Images obtained from a fluorescence microscope (Leika, Germany) were analyzed using Image J. The colony formation assay was used to evaluate the cell clonogenic ability. The transfected Huh7 and MHCC-LM3 cells were seeded in a 35mm-diameter petri dish and cultured for up to 14 days, respectively. Cell colonies were fixed with 4% paraformaldehyde and stained with 0.1% crystal violet (Beyotime) for 20 minutes, the colonies were counted under a light microscope.

#### 2.8.4 Transwell Assay

Cell invasion was evaluated by performing the Chamber matrigel invasion 24-well units (Costar) according to the manufacturer’s instructions. The transfected cells were suspended in a serum-free medium and plated into the upper chamber of the transwell system with a pore size of 8 µm. The bottom chamber was filled with a medium containing 10% FBS. After incubation for 24 h, the migrated/invaded cells in the lower chamber (below the filter surface) were fixed in 4% paraformaldehyde, stained with crystal violet solution, and counted under a microscope.

#### 2.8.5 Wound Scratch Assay

Wound scratch assays were used to assess the migratory ability of Huh7 and MHCC-LM3 cells *in vitro*. AKR1C3 downregulated Huh7 and MHCC-LM3 cells (including negative control cells) were planted in 3.5 cm dishes and grown until 80%–90% confluent. Then, a 100 μl yellow pipette tip was used to scratch the cell monolayers and the cells were maintained in DMEM-6429 medium. The area of the cell-free wound was measured with microscopy at 0 and 24 h.

#### 2.8.6 CCK-8 Experiments

The CCK-8 assay (Dojindo, Japan) was performed to assess Huh7 and MHCC-LM3 cells proliferation. Cells were seeded at a density of 4×10^3^ cells/well in 96 wells plates, then added 20 µl of CCK-8 reagent to each well of a 96-well plate, and incubated the cells for 2 h at 37°C. At 6, 24, 48, 72, and 96 h, cell viability was detected by scanning with a microplate reader (Tecan, Switzerland) at 450 nm.

### 2.9 Statistical Analysis

R software 3.6.3 was used for all data management and analyses in present study. Wilcoxon rank-sum test was used to compare the differences of quantitative variables, and Spearman correlation test was used to explore the correlation between variables. Schoenfeld residuals test was performed to test the proportional hazard assumption of Cox regression model. All the statistical tests were two-sided, and *p* < 0.05 were considered to be statistically significant.

## 3 Results

### 3.1 Survival-Related eRNAs, eRGs and Prognostic Signature

**Figure 1:**
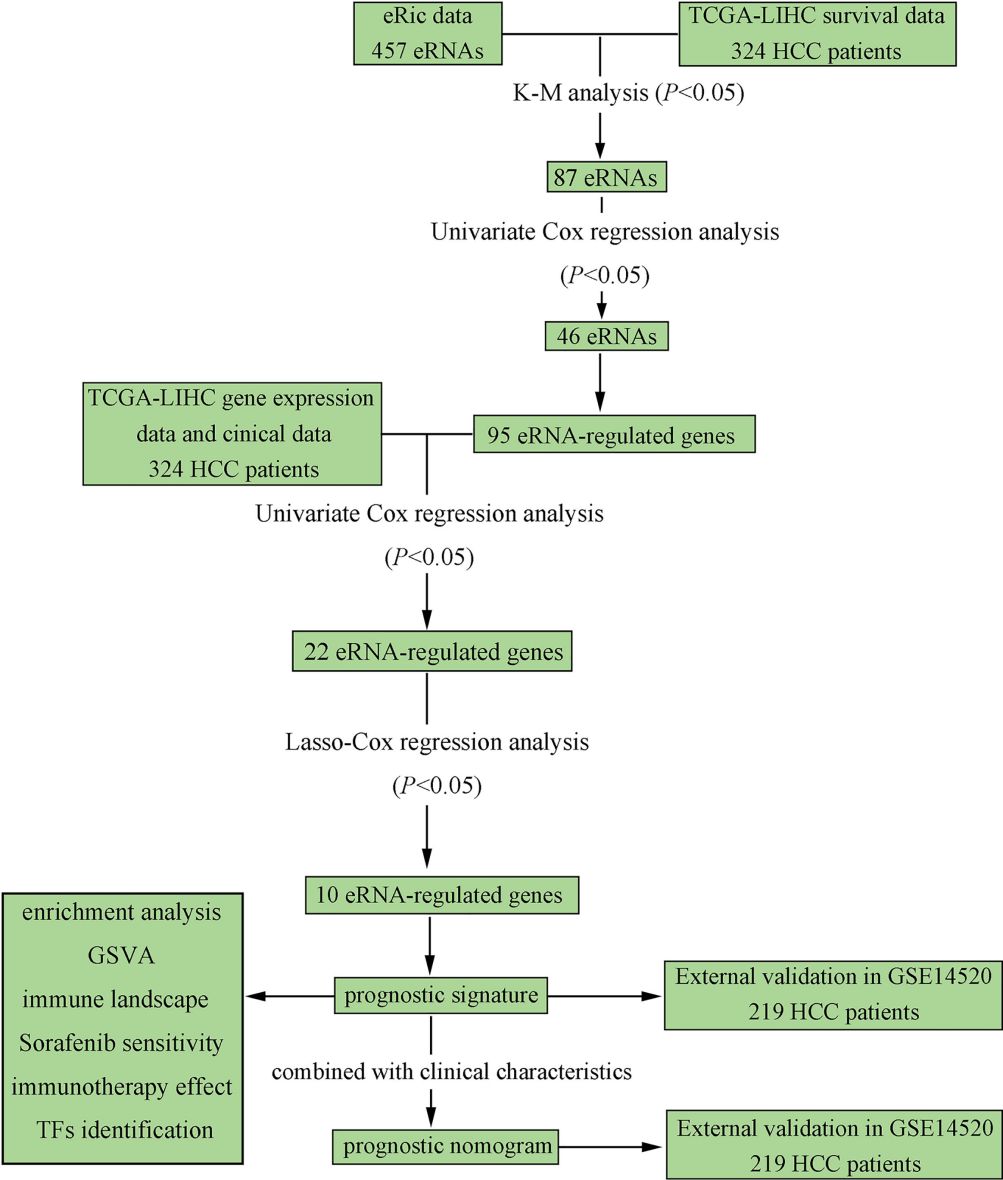
Flowchart elaborating the scheme of construction and validation of prognostic signature and nomogram based on eRGs for HCC patients.

The overall flow chart of our study is depicted in **Figure 1**. A total of 324 HCC patients from TCGA-LIHC were included as the training set, and 219 HCC patients from GSE14520-GPL3921 were used as the external validation set. The general clinical characteristics of the two datasets are exhibited in **Supplementary Table S1**.

eRNA expression profile data of 324 TCGA-LIHC patients were downloaded from the eRic data portal, and 457 eRNAs were obtained for K-M analysis and univariate Cox regression. Based on the results of survival analysis, 46 eRNAs were identified that were significantly associated with overall survival (*p* < 0.05). Then 95 target genes of the survival-related eRNAs were obtained by referring to the eRNA target genes list from the eRic database. The survival-related eRNAs and their target genes list are shown in **Supplementary Table S2**.

**Figure 2:**
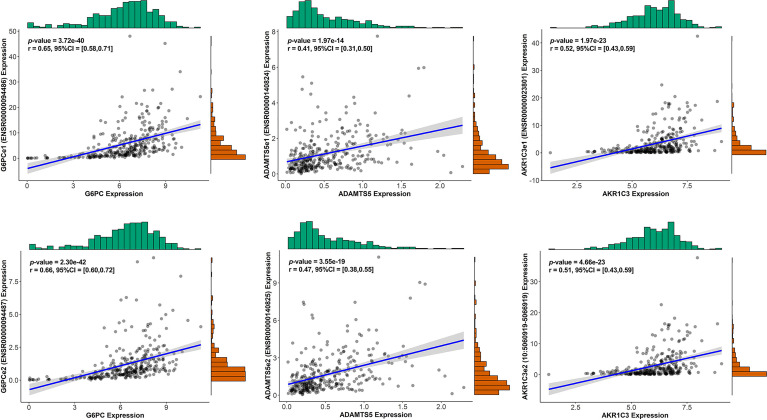
The correlation between genes in the signature and their corresponding eRNA (part results).

We conducted univariate Cox regression analysis on survival-related eRNAs target genes in training set, and 22 eRGs were significantly associated with overall survival (*p* < 0.05). After that, Lasso-Cox regression analysis was carried out to discover eRGs related to survival and construct a prognostic signature, which composed of 10 genes, including SSRP1, SSB, IGFBP4, SUOX, RDH16, G6PC, AKR1C3, NUP205, ADAMTS5, and RRAGD, and the correlation between these genes and their corresponding eRNAs are all significant (correlation coefficients larger than 0.3, *p* < 0.0001). Part of the results are shown in **Figure 2**, and the horizontal coordinate indicates the expression of genes in the signature, the vertical coordinate indicates the expression of their corresponding eRNAs. Other results have been shown in **Supplementary Figure S1**.

**Figure 3:**
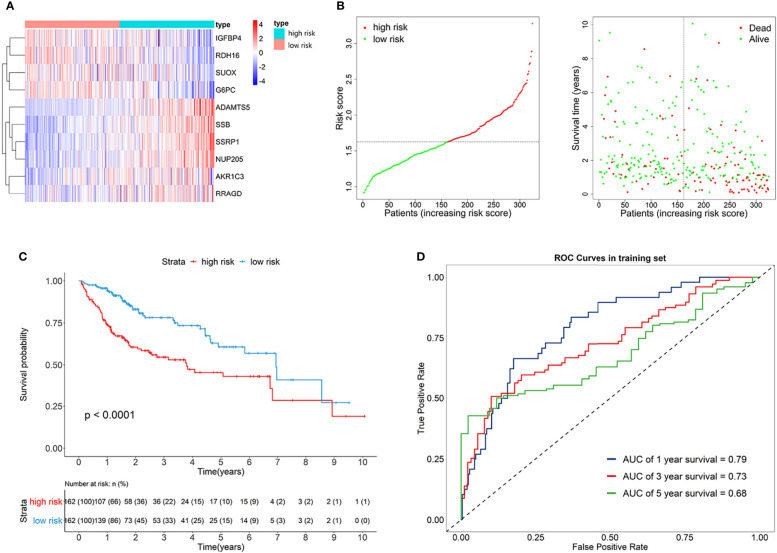
**(A)** Heatmap of the signature genes expression profiles in training set, and the colors represent centered and scaled log2FPKM value in the row direction (FPKM is defined as fragments per kilobase of transcript per million mapped reads); **(B)** Survival status distribution of patients in high risk and low risk group; **(C)** Kaplan-Meier curves of the signature for high risk and low risk group in training set; **(D)** Time-dependent ROC curves of the signature for 1-, 3-, 5-year overall survival in training set.

The risk score of each patient can be calculated by using the following formula based on the corresponding coefficients of genes: *riskScore*  =  0.2209  ×  *Exp*_SSRP1_ + 0.1789  ×  *Exp*_SSB_  +  (−0.0131)  ×  *Exp*_IGFBP4_  +  (−0.0522)  ×  *Exp*_SUOX_  +  (−0.0053)  ×  *Exp*_RDH16_  +  (−0.0356)  ×  *Exp*_G6PC_  +  0.0563  ×  *Exp*_AKR1C3_  +  0.0106  ×  *Exp*_NUP205_  +  0.4235  ×  *Exp*_ADAMTS5_  +  0.0469  ×  *Exp*_RRAGD_ . Subsequently, we obtained all the risk scores of patients in the TCGA dataset and considered the median as the cutoff point to divide the patients into high- and low-risk groups. **Figure 3A** presents the expression profiles of 10 survival-related eRGs in the signature, and the expressions of 10 genes were all significantly different between the two groups (**Supplementary Figure S2**). Among them, the expressions of IGFBP4, SUOX, RDH16, and G6PC were lower in the high-risk group than those in the low-risk group, while the expressions of SSRP1, SSB, NUP205, AKR1C3, ADAMTS5, and RRAGD were higher in high-risk group. **Figure 3B** shows risk scores and survival status in the two groups. The Kaplan-Meier survival curves in the training set are shown in **Figure 3C**, which revealed that patients in the high-risk group had a significantly worse prognosis than those in the low-risk group (*p* < 0.0001). The time-dependent ROCs for 1-, 3-, and 5-year OS are exhibited in **Figure 3D**, and their AUCs (area under the ROC curve) were 0.79, 0.73, and 0.68, respectively. The C-index (concordance index) of the signature was 0.70. All the above results demonstrated that the signature composed of 10 eRGs poses a good prognostic performance.

**Figure 4:**
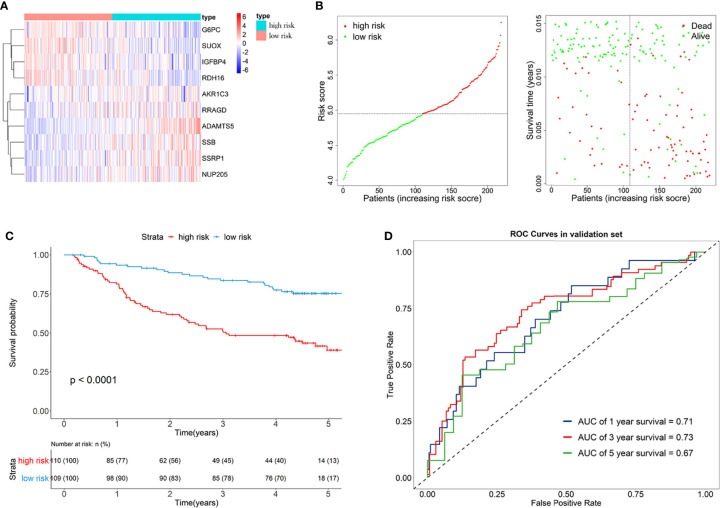
**(A)** Heatmap of the signature genes expression profiles in validation set, and the colors represent centered and scaled log2FPKM value in the row direction (FPKM is defined as fragments per kilobase of transcript per million mapped reads); **(B)** Survival status distribution of patients in high risk and low risk group; **(C)** Kaplan-Meier curves of the signature for high risk and low risk group in validation set; **(D)** Time-dependent ROC curves of the signature for 1-, 3-, 5-year overall survival in validation set.

Furthermore, the GSE14520 dataset was considered as the external validation data to confirm the performance of the eRGs signature. The expression of genes, risk scores, and survival status are shown in **Figures 4A, B**. The Kaplan-Meier survival curves were also significantly different (*p* < 0.0001) between the two different risk groups (**Figure 4C**). The AUCs of time-dependent ROCs for 1-, 3-, and 5-year OS were 0.71, 0.73, 0.67, respectively (**Figure 4D**), and the C-index was 0.68. Taken together, these results suggested that the signature had a good capability of predicting survival.

### 3.2 Prognostic Nomogram

**Figure 5:**
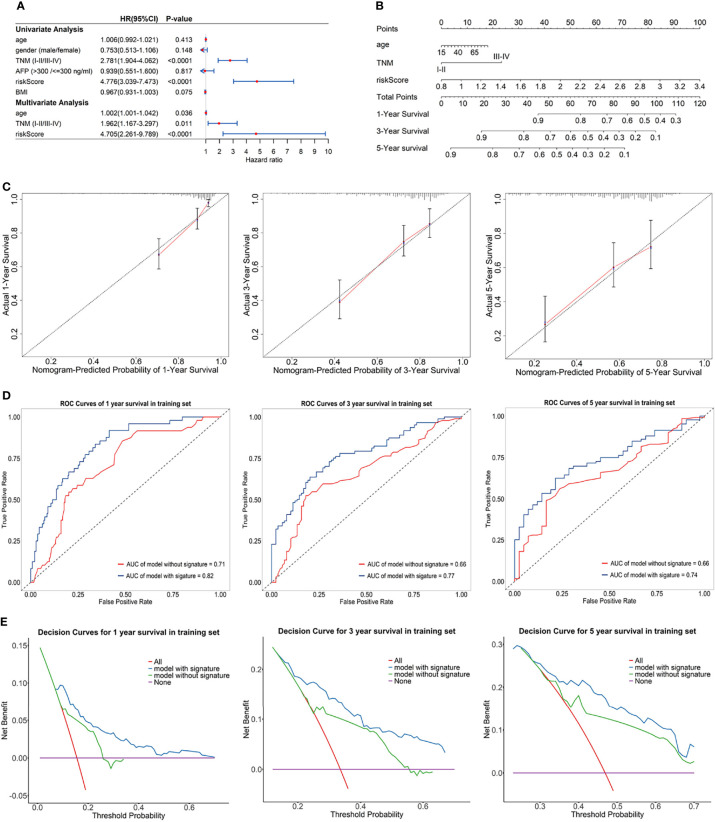
**(A)** Forest plot for univariate Cox regression analysis and multivariate Cox regression analysis; **(B)** Nomogram predicting 1-, 3-, 5-year survival probability; **(C)** Calibration curves of the nomogram for 1-, 3-, 5-year overall survival in training set; **(D)** Time-dependent ROC curves of the nomogram for 1-, 3-, 5-year overall survival in training set; **(E)** DCA curves of the nomogram for 1-, 3-, 5-year overall survival in training set.

Univariate Cox regression analysis and multivariate Cox regression analysis were used to identify the prognostic factors in TCGA training set, and the risk score of the eRGs signature was a crucial independent prognostic predictor (**Figure 5A**). The results of Schoenfeld residuals test can be found in **Figure S3** (the global Schoenfeld test *p* = 0.29), which indicated that the Cox model satisfied the proportional hazard assumption. Then a nomogram containing age, TNM stage, and risk score was established to predict 1-, 3-, and 5-year survival based on the training set (**Figure 5B**). We conducted a series of internal validation on the performance of the nomogram. Calibration curves presented good concordance between the nomogram-predicted survival and the actual survival of 1-, 3-, and 5-year (**Figure 5C**). The C-index of the nomogram was 0.73. Time-dependent ROC curves at 1-, 3-, and 5-year were exhibited in **Figure 5D**, and AUCs of the nomogram at 1-, 3-, and 5-year were 0.82, 0.77, 0.74, respectively. Furthermore, the model with signature had significantly higher AUC values than the model without signature, which suggested that our eRGs signature and nomogram possessed an excellent prognostic performance. DCA curves of the nomogram for 1-, 3-, 5-year survival in training set are shown in **Figure 5E**, which demonstrates that the nomogram had a high net benefit.

The prognostic performance of nomogram was also validated in GEO validation set. Kaplan-Meier survival curves of the nomogram in TCGA training set and GEO validation set were presented in **Supplementary Figures S4A, B**, and the curves also significantly different (*p* < 0.0001) between the high-risk and low-risk group. The C-index of the nomogram in validation set was 0.71. AUCs of the nomogram at 1-, 3-, and 5-year were 0.74, 0.77, 0.74, respectively, and significantly larger than that model without signature (**Supplementary Figure S4D**). In addition, calibration curves, ROC curves, and DCA curves (**Supplementary Figures S4C, E**) in GEO validation set were all further confirmation that the nomogram had a good predictive value and clinical application value.

### 3.3 Pathways and Mechanism Analyses

**Figure 6:**
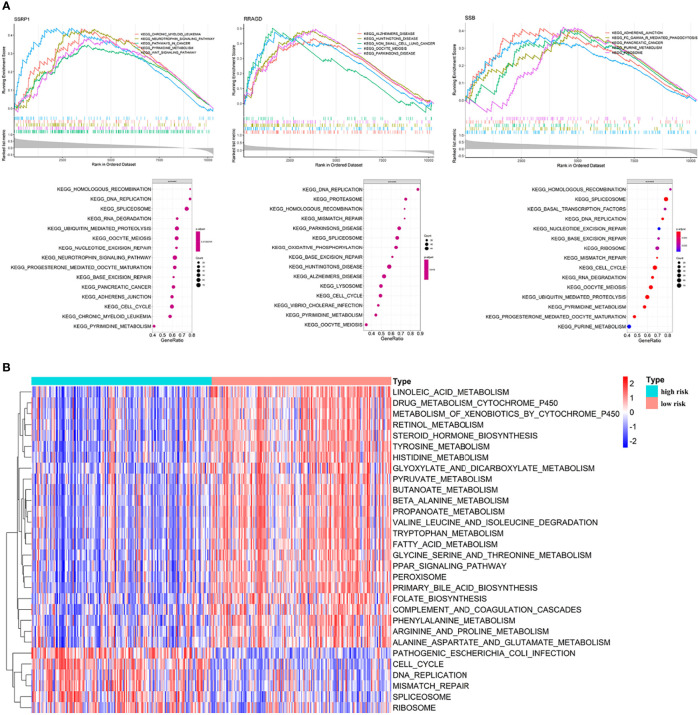
Enrichment analyses. **(A)** Single gene GSEA results; **(B)** GSVA results: different pathways between high risk patients and low risk patients.

We performed GO and KEGG enrichment analysis on the survival-related eRGs and identified 28 GO terms and two KEGG pathways (**Supplementary Table S3** and **Figure S5**). These survival-related eRGs were enriched in steroid metabolic process, lipid transport, carbohydrate catabolic process, coenzyme metabolic process, oxidoreductase activity, and other GO terms. The enriched KEGG pathways were ABC transporters and steroid hormone biosynthesis. The single gene GSEA results can be seen in **Figures 6A, B**, **Supplementary Figure S6** and **Supplementary Table S4** exhibit the different pathways between the high-risk and low-risk group. The up-regulated pathways in high-risk patients were pathogenic Escherichia coli infection, cell cycle, DNA replication, mismatch repair, spliceosome, and ribosome. In comparison, the down-regulated pathways in high-risk patients were fatty acid metabolism, drug metabolism cytochrome P450, steroid hormone biosynthesis, primary bile acid biosynthesis, PPAR signaling pathway, complement and coagulation cascades, amino acid metabolism, linoleic acid metabolism, etc.

### 3.4 Immune Landscape

eRGs we identified were associated with immune function, and G6PC, SSRP1, NUP205, ADAMTS5, and RRAGD were all related to immune response or process, so we further explored the relationship between risk scores and immune landscape.

**Figure 7:**
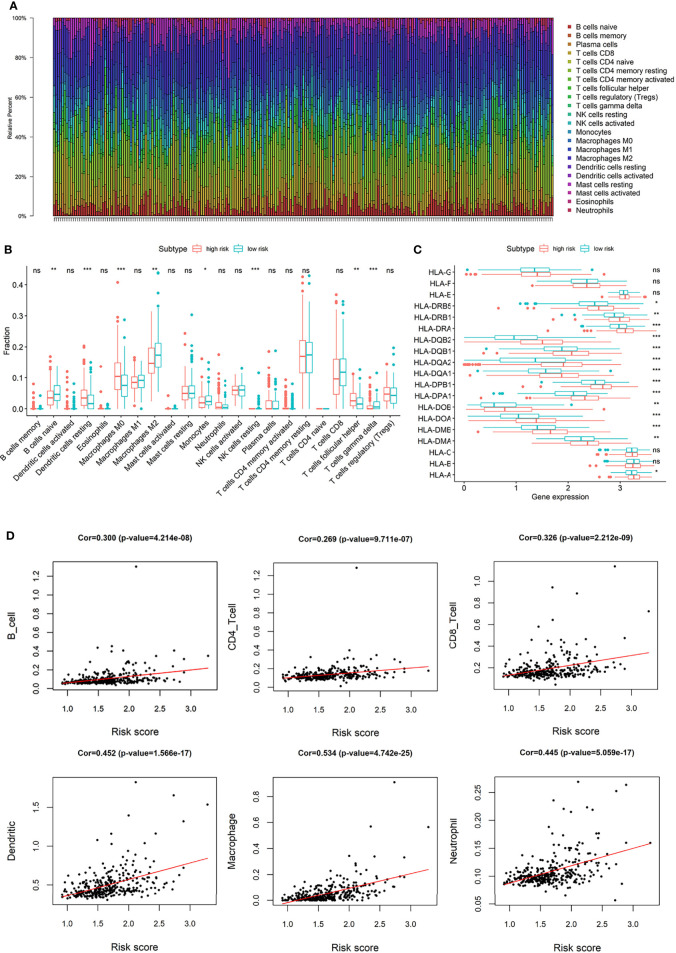
**(A)** The composition of immune infiltrating cells in TCGA-LIHC patients; **(B)** Differences of immune infiltration cells between high and low risk group; **(C)** Differences of HLA-related genes between high and low risk group; **(D)** The correlation between risk score and the abundances of B cells, CD4+ T cells, CD8+ T cells, macrophages, neutrophils, and dendritic cells. (*p < 0.05; **p < 0.01; ***p < 0.001; “ns” means “no significant difference”).

Macrophages and T cells had a large proportion in the immune infiltration of TCGA HCC patients (**Figure 7A**). The compositions of immune infiltration were significantly different between the two risk groups. The proportions of B cells naïve, Macrophages M2, Monocytes, NK cells resting, T cells gamma delta were lower in the high-risk group than those in the low-risk group, while dendritic cells resting, Macrophages M0, T cells follicular helper had higher proportions in the high-risk group (**Figure 7B**). The abundances of B cells, CD4+ T cells, CD8+ T cells, macrophages, neutrophils, and dendritic cells were estimated based on Timer algorithm, and they were all significantly correlated with risk score (Spearman correlation test, *p* < 0.0001, **Figure 7D**). In addition, it can be seen from **Figure 7C** that, except for HLA-B, HLA-C, HLA-E, HLA-F, and HLA-G, the expression levels of HLA-related genes were significantly different between the two groups with a higher expression in the high-risk group.

### 3.5 Drug Sensitivity, Immunotherapy Effect and Transcription Factors

**Figure 8:**
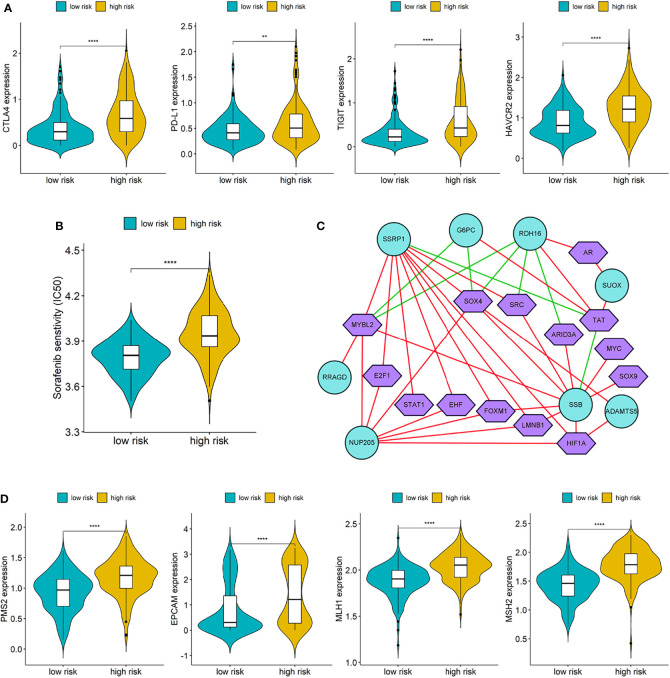
**(A)** Differences of immune checkpoints between high risk group and low risk group; **(B)** Difference of Sorafenib sensitivity (IC50) between high risk and low risk patients; **(C)** The network diagram of the correlation between TFs and genes in the prognostic signature. Green circles represent genes in the signature and purple polygons represent TFs. The red lines indicate positive correlation and the green lines indicate negative correlation; **(D)** Differences of DNA damage repair related genes between high and low risk group. (**p < 0.01; ****p < 0.0001).

The expressions of four common immune checkpoints (CTLA4, PD-L1, TIGIT, and HAVCR2) were all significantly lower in the low-risk group than high-risk group (*p* < 0.05), which indicated that low-risk patients may have better outcomes when treated with immune checkpoint inhibitors (**Figure 8A**). There was a significant difference in IC50 of Sorafenib between the two different risk groups, and low-risk patients have a lower IC50, which suggested that patients in the low-risk group may be more sensitive to Sorafenib (**Figure 8B**).

We downloaded a list of 318 TFs from Cistrome and identified 14 TFs related to the genes in prognostic signature (**Supplementary Table S5**). The network diagram of the correlation between TFs and genes is shown in **Figure 8C**, and SSRP1, SSB, NUP205, and RDH16 have more co-expressed TFs with positive correlations. Furthermore, we also compared the differences of DNA damage repair related genes (PMS2, EPVAM, MLH1, and MSH2) between the high-risk group and low-risk group, and the expressions of these genes were all significantly higher in the high-risk group than low-risk group (**Figure 8D**).

### 3.6 Experimental Confirmation of AKR1C3 *In Vitro* on Two HCC Cell Lines

**Figure 9:**
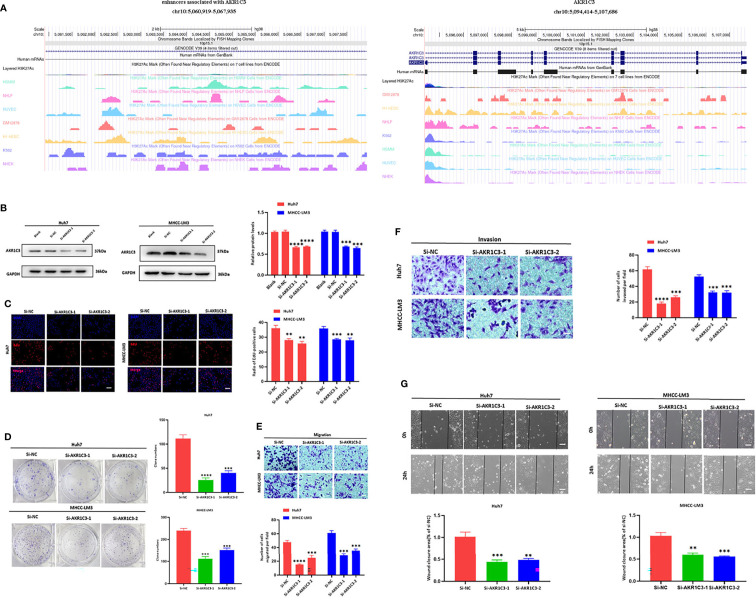
**(A)** The locations of AKR1C3 and enhancers associated with AKR1C3 on chromosome. **(B)** Western blot analysis to examine the efficiency of the AKR1C3 knockdown. **(C)** Proliferation ability in AKR1C3 knockdown Huh7 and MHCC-LM3 cells by EdU staining. **(D)** Colony-forming abilities in AKR1C3 knockdown Huh7 and MHCC-LM3 cells by clonogenic assays. **(E, F)** Transwell assays to detect the migration and invasive capacities in AKR1C3 knockdown Huh7 and MHCC-LM3 cells. **(G)** Wound-healing assay was performed to measure the migration ability of various cells as indicated. Magnification, × 200 **(C, E, F)**, × 40 **(G)**. Scale bar, 100 mm **(C, E, F)**, 500 mm **(G)**. Data were shown as mean ± SD of at least three independent experiments. (**p < 0.01; ***p < 0.001; ****p < 0.0001).

**Figure 10:**
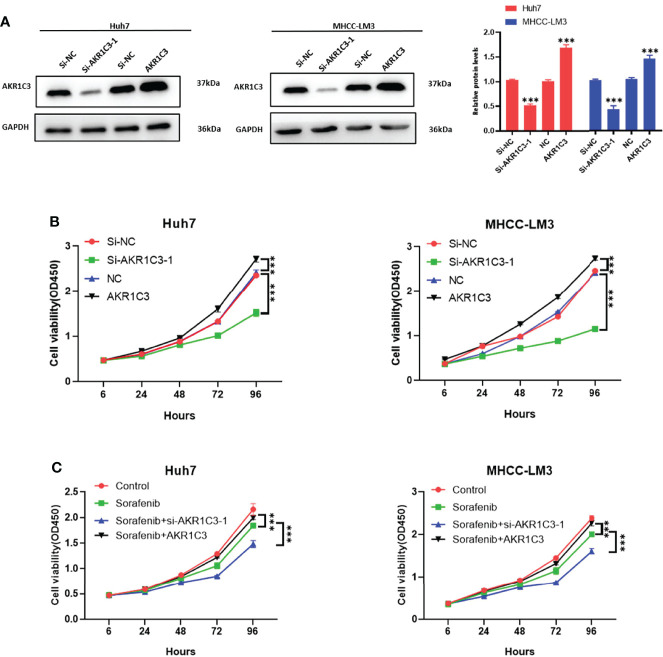
**(A)** Western blot analysis to examine the efficiency of the AKR1C3 knockdown and overexpression. **(B, C) **Proliferation curves were determined in AKR1C3 knockdown and overexpression Huh7 and MHCC-LM3 cells by cell counting kit-8 (CCK-8) assays in normal culture conditions and sorafenib sensitivity experiment. Data were shown as mean ± SD of at least three independent experiments. (***p < 0.001).

We utilized the UCSC Genome Browser (http://genome.ucsc.edu/index.html) visualized the location of AKR1C3 and relative enhancers (**Figure 9A**). The AKR1C3 located on chromosome 10:5094414-5107686, and the enhancers associated with AKR1C3 located on chromosome 10:5060919-5067935. After transfection with AKR1C3-specific siRNA in Huh7 and MHCC-LM3 cell lines, both of the two selected siRNAs could significantly decrease AKR1C3 expression compared with control cells according to the western blot analysis (**Figure 9B**). EdU staining and colony formation assays were applied to assess the effect of si-AKR1C3-transfection on proliferation. The results indicated that compared with the control (si-NC), the si-AKR1C3 significantly reduced cell viability (**Figure 9C**) and the number of colony formations (**Figure 9D**). We further explored the effects of AKR1C3 on migration and invasion capacity of Huh7 and MHCC-LM3 cells *via* Transwell chamber assays. The migration and invasion abilities were significantly inhibited in si-AKR1C3-1 and si-AKR1C3-2 groups compared to the si-NC group (**Figures 9E, F**). Consistently, the wound healing assay revealed that the si-NC group had a higher efficiency at closing the wound width than those in the AKR1C3 silencing group (**Figure 9G**). AKR1C3 overexpression promoted and silencing inhibited protein expression (**Figure 10A**) and cell proliferation (**Figure 10B**). In comparison with the control group and sorafenib groups, the si-AKR1C3-1 plus sorafenib groups showed significantly decreased cell proliferation rate, then it is of note that the AKR1C3 plus sorafenib groups further enhanced cell proliferation rate compared with the sorafenib groups (**Figure 10C**). Altogether, these experimental results suggested that the knockdown of AKR1C3 inhibited the cell proliferation, migration, and invasion in HCC cell lines, which indicated that AKR1C3 plays a key role in HCC cell proliferation and aggressiveness.

## 4 Discussion

Enhancer RNAs (eRNAs) regulate the expression of oncogenes or tumor suppressors and play a prominent role in the tumorigenesis, progression, and proliferation of cancers, which could be used as potential and valuable diagnostic, prognostic, and therapeutic markers for cancers. Considering this, we attempted to establish a novel and robust signature based on eRGs to provide a new perspective for prognostic prediction and optimization of personalized treatment in HCC patients for the first time.

In this study, we identified a signature including 10 survival-related eRGs (SSRP1, SSB, IGFBP4, SUOX, RDH16, G6PC, AKR1C3, NUP205, ADAMTS5, and RRAGD). The signature stratified HCC patients into high- and low-risk groups, and patients in the high-risk group significantly tended to have a poorer prognosis. Previous studies have already constructed different signatures for predicting the overall survival in HCC patients (41–43). The comparison of the AUCs for 1-, 3-, 5-year overall survival of our eRGs signature and those of previous signatures were presented in **Table 1**. Obviously, our signature has a better prognostic value than others, especially in external validation dataset, which indicates that our signature is more robust. Furthermore, we found that the signature was an independent prognostic factor, which can combine age and TNM stage to establish a nomogram. The calibration curves of the nomogram indicated that the predicted outcomes were in good agreement with the actual outcomes in training set and validation set. Furthermore, time-dependent ROC curves exhibited that the sensitivity and specificity of model with signature were significantly improve compared with those of the model with signature. According to the nomogram, clinicians can predict the prognosis of HCC patients and provide appropriate individualized treatment to improve their quality of life.

**Table 1:** Comparison of AUCs for prognostic signatures in different studies.

	Our study	Zhang et al. (2020) (41)	Li et al. (2017) (42)	Zhang et al. (2020) (43)
**Signature**	10-genes	14-genes	3-genes	8-genes
**Training set**	TCGA (n = 324)	TCGA (n = 312)	TCGA (n = 360)	TCGA (n = 361)
1-year AUC	0.79	0.71	0.73	0.77
3-year AUC	0.73	0.74	0.71	0.75
5-year AUC	0.68	0.64	0.69	0.75
**Validation set**	GSE14520 (n = 219)	GSE14520 (n = 225)	GSE14520 (n = 209)	GSE14520 (n = 221)
1-year AUC	0.71	0.64	0.65	0.66
3-year AUC	0.73	0.59	0.62	0.66
5-year AUC	0.67	0.65	0.62	0.67

Genes in the signature and their corresponding eRNAs were all significantly correlated. Among them, ENSR00000052553 is the cancer-type-specific eRNA of HCC, which regulates the expression of SUOX and RDH16, and can be considered as a potential target eRNA for further treatment studies of HCC. In addition, G6PC, AKR1C3, and ADAMTS5 were all regulated by two neighbor eRNAs, which indicated that the enhancers at those locations may cluster into super-enhancers. Studies have already demonstrated that super-enhancers play a significant role in oncogene activation, process of tumorigenesis, and tumor cell proliferation (23–25), so our findings may lay the groundwork for investigating the mechanism of super enhancers in HCC.

Compared with the low-risk group, four genes (IGFBP4, SUOX, RDH16, and G6PC) of the prognostic signature downregulated in the high-risk group, while the other six genes (SSRP1, SSB, NUP205, AKR1C3, ADAMTS5, and RRAGD) upregulated conversely. IGFBP4 is the smallest member of human insulin-like growth factors binding proteins (IGFBPs) (44), which is involved in the inhibition of oncogenic pathways and exerts a powerful tumor suppressor function in HCC cells. It also had been demonstrated that low expression of IGFBP4 is associated with poor prognosis in HCC patients (45). SUOX was found to decrease with the progression of HCC and considered as an independent prognostic factor for overall survival and time to recurrence in HCC patients (46). RDH16 is a tumor-suppressing gene, and it had been reported that downregulation of RDH16 occurs in approximately 90% of primary HCC patients with poor prognosis (47). In addition, RDH16 was also contained in a robust twelve-gene signature for predicting survival of HCC patients (48). The deficiency of G6PC can cause glycogen storage disease type Ia (GSD-Ia), which may lead to HCC (49). A previous study has identified G6PC as a potential prognostic target in clear cell renal cell carcinoma, and its low expression associated with poor survival and aggressive progression (50). SSRP1 can regulate the proliferation and metastasis of HCC, its aberrant overexpression is related to higher serum AFP level, larger tumor size, and higher T stage of HCC patients. It has been considered as a prognostic biomarker associated with CD8+ T cell infiltration in HCC, and patients with higher expression of SSRP1 have shorter overall survival and faster recurrence (51, 52). SSB plays a significant role in DNA replication (53), its overexpression may promote the proliferation of HCC cells. Xiong et al. suggested that the upregulation of NUP205 correlated with severe TNM stage and poor survival, which demonstrated that it can be seen as a biomarker for prognostic prediction in HCC patients (54). It was found that higher ADAMTS5 expression had a significant association with development and poorer survival of HCC, and its impact on prognosis was specific for HCC among other cancer types from TCGA project (55). Furthermore, ADAMTS5 was also identified as a prominent gene in a hypoxia-related and immune-associated prognosis signature for HCC (56). RRAGD can promote cell proliferation, invasion, migration, aerobic glycolysis, and Warburg effect (an important characteristic of cancer cell metabolism) of HCC. Upregulation of RRAGD is associated with poor prognosis (57).

AKR1C3 consisted of 323 amino acids with a predicted molecular weight of 36,853 Da Like other AKR enzymes it is a soluble monomeric NAD(P)(H) dependent oxidoreductase, the enzyme that converts carbonyl groups into secondary alcohols (58). Overexpression of AKR1C3 is usually associated with prostate cancer progression, aggressiveness, and resistance to AR-targeted therapies (59). According to a previous clinical study, upregulation of AKR1C3 is an indicator of poor prognosis in HPV16-associated and HPV-negative oropharyngeal squamous cell carcinoma (OPSCC) (60). The results from our bioinformatics analysis were confirmed by a series of experiments, and we found that the silence of AKR1C3 in Huh7 and MHCC-LM3 cells can significantly inhibit HCC cell viability, clone formation, migration, invasion ability, and wound closure potential. In addition, AKR1C3 may be regulated by super-enhancers based on our study. All the above indicated that AKR1C3 is an important eRG related to the progression and prognosis of HCC, which may be a potential biomarker for HCC treatment and intervention.

The enrichment results presented that survival-related eRGs were enriched in ATPase activity, lipid transporter activity, steroid hormone biosynthesis, and ABC transporters, etc. GSVA identified a number of significant pathways that may affect prognostic outcomes of HCC patients. The downregulation of fatty acid metabolism, drug metabolism cytochrome P450, steroid hormone biosynthesis, primary bile acid biosynthesis, complement and coagulation cascades, PPAR signaling pathway, and the upregulation of cell cycle, DNA replication may be the mechanism for poorer prognosis of HCC. Therefore, our enrichment results provide new insights into the deeper investigation of molecular mechanisms and the development of targeted drugs for HCC patients.

Immune microenvironment plays an important role in the occurrence and development of tumors, which has attracted attentions of researchers. According to our results, immune infiltration and HLA may be considered important factors in exploring the specific mechanism and improving the outcomes of HCC patients. Immune checkpoints have received a great deal of attention in cancer treatment in recent years. We checked the differences of expression of immune checkpoints (CTLA4, PD-L1, TIGIT, and HAVCR2) between the high-risk group and low-risk group, and found that they all had higher expression in the high-risk group, which was in line with previous study (61).

Although Sorafenib is the standard systemic therapeutic agent available in HCC patients, the mechanism of drug resistance and the existence of heterogeneity make patients have different drug treatment effects. So, we estimated IC50 of Sorafenib in the two groups and found that the low-risk patients performed better to Sorafenib, which may contribute to the efficient and rational medication of HCC patients. Co-expression analysis of genes and TFs showed that SSRP1 and SSB, NUP205 had positive correlations with their co-expression TFs, while G6PC and RDH16 were negatively correlated with their co-expression TFs. Meanwhile, expression of SSRP1, SSB, and NUP205 were all positively correlated with risk score, and expression of G6PC and RDH16 were negatively correlated with risk score. These findings suggested that eRNAs may bind to these TFs to regulate the expression of genes in the prognostic signature, which seemed to shed light on the investigation of HCC pathological mechanism and therapeutic targets.

Taken together, some advantages of our study deserve to be underscored. First, we constructed prognostic signature and nomogram for HCC based on eRGs for the first time, and the model performed a better predictive ability and provided a novel direction for the underlying pathological mechanism of HCC. Second, we identified survival-related eRNAs directly in HCC patients rather than select them from differential expressed eRNAs between patients and normal controls, so that more comprehensive information can be considered. Third, signature composed of specific genes is more economical and practical than whole-genome sequencing, and easy to routinely test. Fourth, the visualization of the nomogram is more convenient to assist clinicians in predicting patients’ prognosis and customizing individualized treatment plans. Last but not least, we confirmed the role of AKR1C3 in the progression and invasion of HCC through a series of *in vitro* experiments. Nonetheless, some limitations still exist in this study. Firstly, the external validation set was also from a public database, and a multicenter cohort study is needed to further prove the generalizability of the models. Secondly, only one eRG in the signature was experimentally verified in the present study, and other eRGs should be validated in the future studies. Thirdly, the regulatory relationships between eRNAs and their target genes from the eRic database used in our study were generated based on data-driven correlations, and their biological regulatory relationships need to be confirmed by rigorous experiments. Finally, multi-omics data such as methylation, long non-coding RNA, and proteomics should be integrated and analyzed to comprehensively elucidate biological regulatory networks.

## 5 Conclusion

Our study has been the first attempt to identify a prognostic signature composed of eRGs for HCC. We also constructed a nomogram incorporating the signature and clinical characteristics to predict overall survival of HCC patients accurately and robustly, which has been validated in external dataset. The signature and nomogram both performed good prognostic ability. AKR1C3 may be a potential biomarker for HCC treatment and intervention through a series of *in vitro* experiments. The findings of this study have significant practical implications in terms of providing a deeper insight into the investigation of pathogenesis of HCC, optimizing individualized treatment, and improving the prognosis of HCC patients.

## Data Availability Statement

The datasets presented in this study can be found in online repositories. The names of the repository/repositories and accession number(s) can be found below: The Cancer Genome Atlas (TCGA) (https://portal.gdc.cancer.gov/); enhancer RNA in cancers (eRic) (https://hanlab.uth.edu/eRic/); Gene Expression Omnibus, GSE14520 -GPL3921.

## Author Contributions

Conceptualization, WZ and WT; methodology, WZ and KC; software, WZ; validation, QiZ; formal analysis, WZ and WT; investigation, LS and YW; resources, KC; data curation, WZ and KC; writing—original draft preparation, WZ and KC; writing—review and editing, QiuZ; visualization, WZ; supervision, QiuZ; project administration, LS and YW; funding acquisition, ML. All authors have read and agreed to the published version of the manuscript.

## Funding

This work was supported by the National Science and Technology Major Project (2016ZX08011005), the National Natural Science Foundation of China (82073666), and the National Natural Science Foundation of China (82003556).

## Conflict of Interest

The authors declare that the research was conducted in the absence of any commercial or financial relationships that could be construed as a potential conflict of interest.

## Publisher’s Note

All claims expressed in this article are solely those of the authors and do not necessarily represent those of their affiliated organizations, or those of the publisher, the editors and the reviewers. Any product that may be evaluated in this article, or claim that may be made by its manufacturer, is not guaranteed or endorsed by the publisher.
