# Supplementary material for: A Novel and Robust Prognostic Model for Hepatocellular Carcinoma Based on Enhancer RNAs-Regulated Genes
Source: Front Oncol. 2022 May 12;12:849242. doi: 10.3389/fonc.2022.849242 (PMC9133429; doi:10.3389/fonc.2022.849242)
Supplement: Supplementary file 1 [file DataSheet_1.docx]

Supplementary Material

# Supplementary Figures and Tables

**Supplementary Table S1.** General clinical characteristics of HCC patients in TCGA and GEO datasets.

|  | **training set** | **external validation set** |
| --- | --- | --- |
|  | **TCGA (*n* = 324)** | **GEO (*n* = 219)** |
| **gender** |  |  |
| female | 101(31.17%) | 30(13.70%) |
| male | 223(68.83%) | 189(86.30%) |
| **age** | 58.81±13.11 | 50.75±10.62 |
| **BMI** | 25.74±6.01 | - |
| **TNM stage** |  |  |
| I-II | 241(74.38%) | 170(77.63%) |
| III-IV | 83(25.62%) | 49(22.37%) |
| **AFP** |  |  |
| >300ng/ml | 60(18.52%) | 98(44.75%) |
| ≤300ng/ml | 190(58.64%) | 118(53.88%) |
| not reported | 74(22.84%) | 3(1.37%) |
| **inflammation** |  |  |
| none | 108(33.33%) | - |
| mild | 90(27.78%) | - |
| severe | 16(4.94%) | - |
| not reported | 110(33.95%) | - |
| **grade** |  |  |
| G1 | 44(13.58%) | - |
| G2 | 155(47.84%) | - |
| G3 | 111(34.26%) | - |
| G4 | 12(3.70%) | - |
| not reported | 2(0.62%) | - |
| **multinodular** |  |  |
| yes | - | 45(20.55%) |
| no | - | 174(79.45%) |
| **cirrhosis** |  |  |
| yes | - | 201(91.78%) |
| no | - | 18(8.22%) |
| **main tumor size** |  |  |
| ≤5cm | - | 139(63.47%) |
| >5cm | - | 79(36.07%) |

**Supplementary Table S2.** List of survival-related eRNAs and survival-related eRNA-regulated genes in HCC patients.

| **survival-related eRNAs** | ENSR00000013279 | | ENSR00000094486 | | ENSR00000140825 | | | ENSR00000240825 | | ENSR00000317983 | |
| --- | --- | --- | --- | --- | --- | --- | --- | --- | --- | --- | --- |
|  | ENSR00000013280 | | ENSR00000094487 | | ENSR00000156919 | | | ENSR00000240826 | | ENSR00000326714 | |
|  | ENSR00000013281 | | ENSR00000094979 | | ENSR00000156920 | | | ENSR00000251054 | | 1:148876276-148882276 | |
|  | ENSR00000023801 | | ENSR00000097601 | | ENSR00000160285 | | | ENSR00000263774 | | 10:5060919-5066919 | |
|  | ENSR00000052553 | | ENSR00000101286 | | ENSR00000160286 | | | ENSR00000264041 | | 11:1669940-1675940 | |
|  | ENSR00000074563 | | ENSR00000101287 | | ENSR00000166220 | | | ENSR00000264109 | | 4:17479163-17485163 | |
|  | ENSR00000080396 | | ENSR00000118679 | | ENSR00000189170 | | | ENSR00000282764 | |  | |
|  | ENSR00000092461 | | ENSR00000121287 | | ENSR00000199892 | | | ENSR00000285337 | |  | |
|  | ENSR00000092462 | | ENSR00000126015 | | ENSR00000218547 | | | ENSR00000292478 | |  | |
|  | ENSR00000094095 | | ENSR00000140824 | | ENSR00000239035 | | | ENSR00000313273 | |  | |
| **survival-related eRNA-regulated genes** | PPIAL4G | CDK12 | | ABCA6 | | ADAMTS5 | BPGM | | GLYAT | | STAG3L3 |
|  | FAM200B | IGFBP4 | | ABCA8 | | MIR155HG | C7orf49 | | OSBP | | STX1A |
|  | ABCA10 | IKZF3 | | ABCA9 | | GAP43 | LUZP6 | | C2orf15 | | PPIAL4G |
|  | AKR1C2 | MED1 | | PRKAR1A | | GAP43 | NUP205 | | INPP4A | | AKR1C2 |
|  | AKR1C3 | WIPF2 | | FAM136A | | AADAC | WDR91 | | MGAT4A | | AKR1C3 |
|  | COQ10A | CD300LG | | SNRPG | | IGSF10 | NIPSNAP3A | | MRPL30 | | FAM99A |
|  | HSD17B6 | G6PC | | PLGLA | | SUCNR1 | C5 | | REV1 | | FAM99B |
|  | RDH16 | CD300LG | | RGPD3 | | AADAC | RAB14 | | TSGA10 | | INS-IGF2 |
|  | SPRYD4 | G6PC | | KLHL23 | | SUCNR1 | STOM | | NEIL3 | | KRTAP5-6 |
|  | SUOX | ACBD4 | | PHOSPHO2 | | PPIAL4G | C5 | | ATOX1 | | LAP3 |
|  | NMB | DCAKD | | SPC25 | | ATOX1 | RAB14 | | GM2A | | QDPR |
|  | WDR73 | NMT1 | | SSB | | GM2A | STOM | | BAZ1B | |  |
|  | ZSCAN2 | PPIAL4G | | ADAMTS5 | | RRAGD | SSRP1 | | POM121 | |  |
|  | USP22 | ABCA5 | | MIR155HG | | AGBL3 | GLYATL1 | | SPDYE7P | |  |


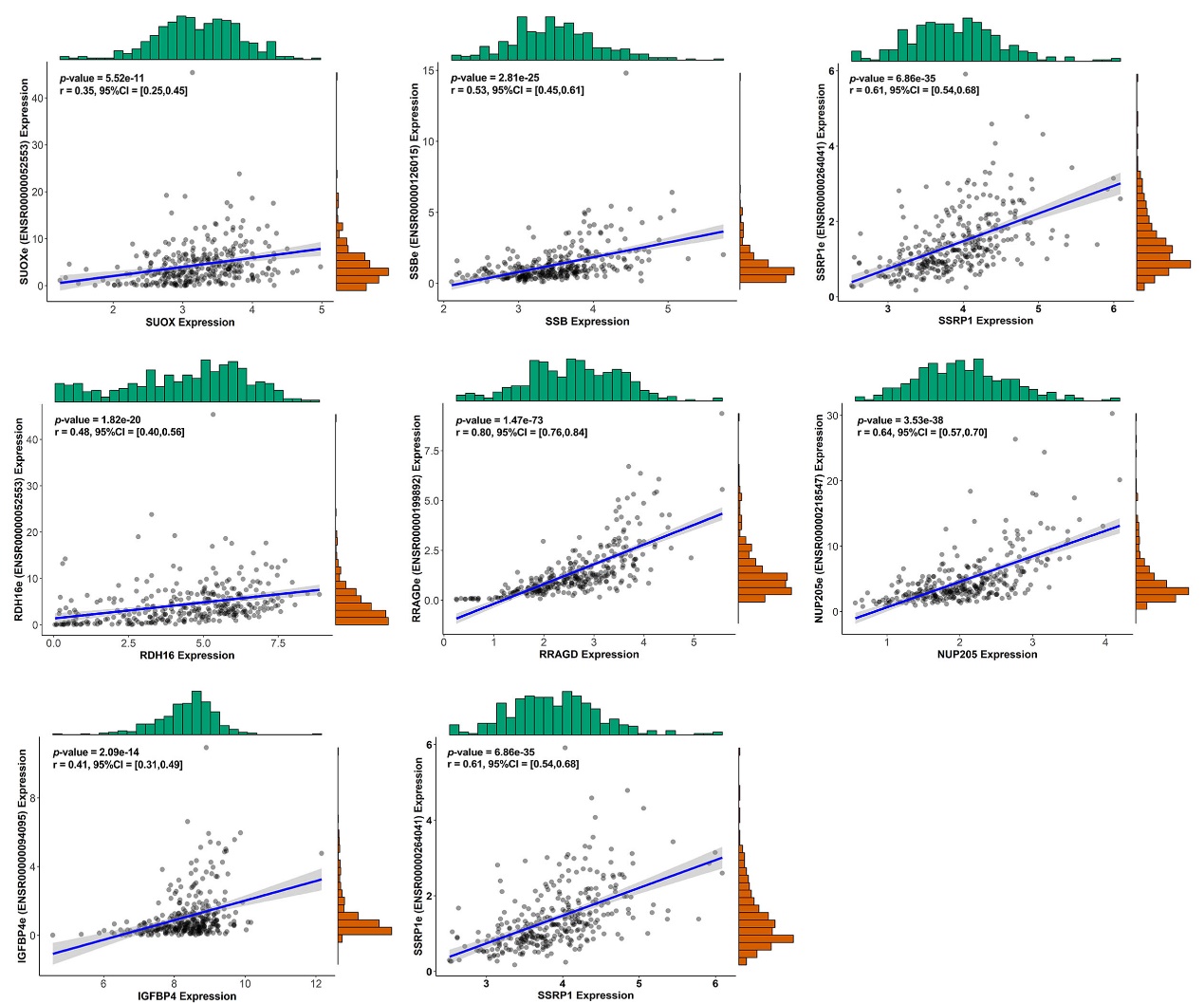


**Supplementary Figure S1.** The correlation between genes in the signature and their corresponding eRNA.


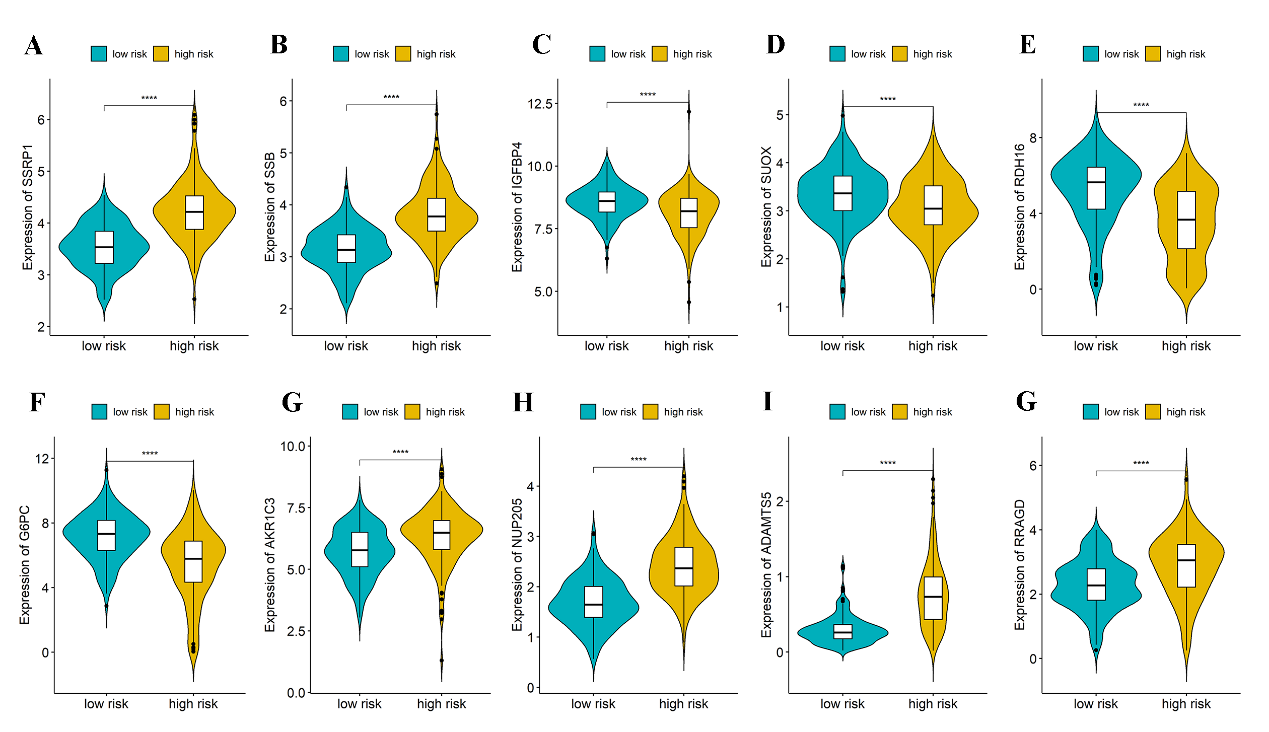


**Supplementary Figure S2.** Different expression of 10 genes in the prognostic signature between high risk group and low risk group (Wilcoxon test). (**p* < 0.05, ***p* < 0.01, ****p* < 0.001, *****p* < 0.0001).


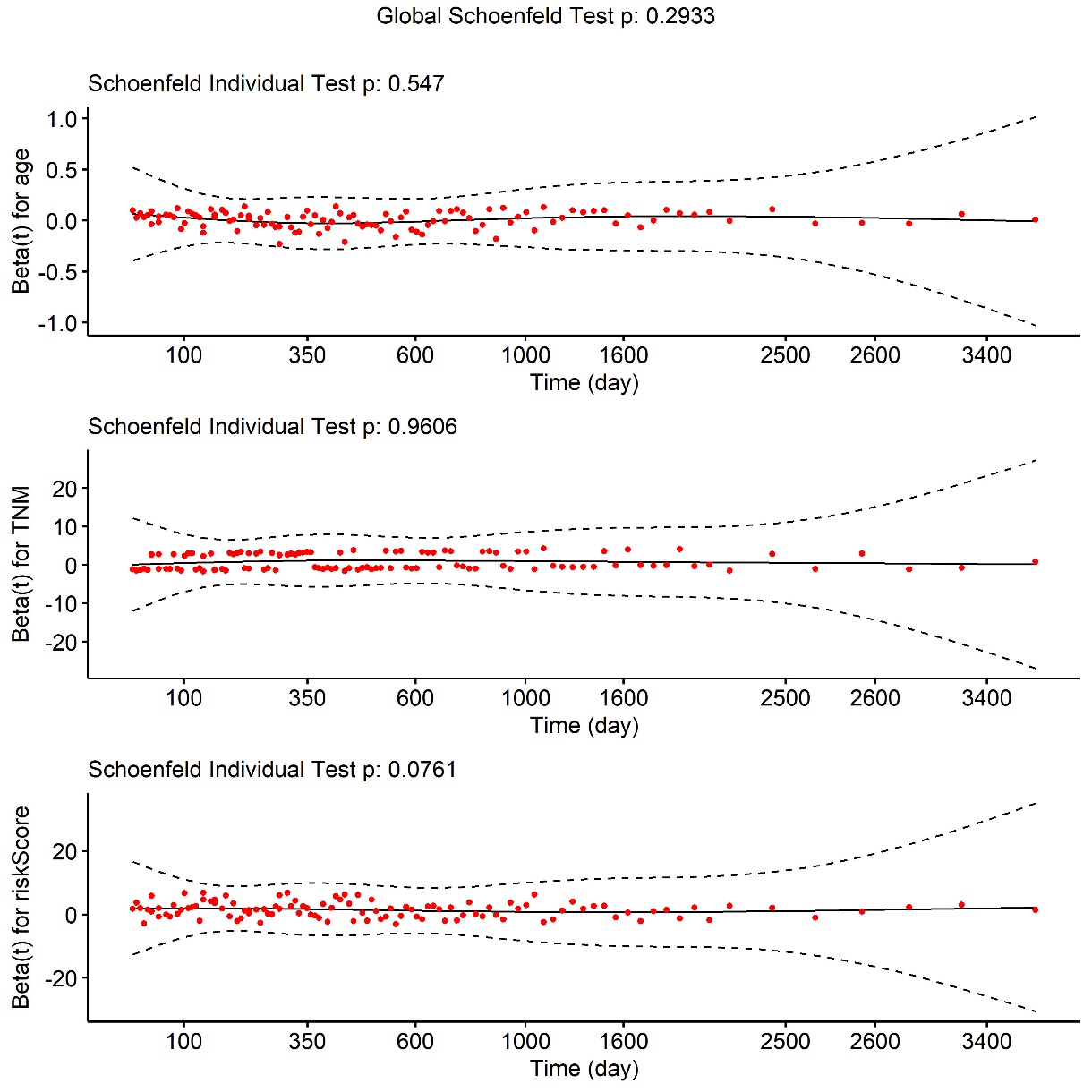


**Supplementary Figure S3.** Schoenfeld residuals test for proportional hazard assumption of Cox regression model.


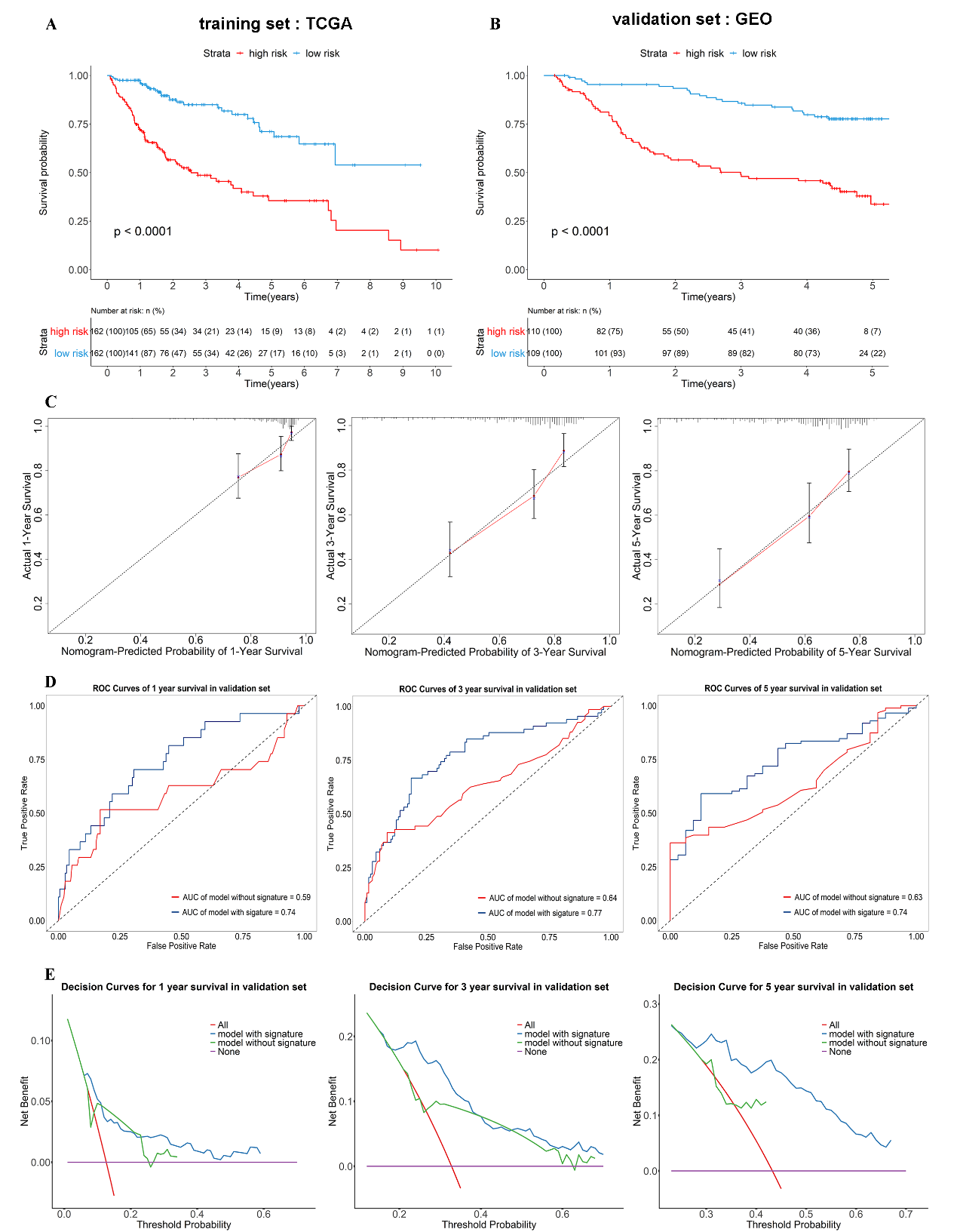


**Supplementary Figure S4.** (A-B) Kaplan-Meier curves of the nomogram for high risk and low risk group in training set and validation set; (C) Calibration curves of the nomogram for 1-, 3-, 5-year overall survival in validation set; (D) Time-dependent ROC curves of the nomogram for 1-, 3-, 5-year overall survival in validation set; (E) DCA curves of the nomogram for 1-, 3-, 5-year overall survival in validation set.

**Supplementary Table S3.** GO and KEGG enrichment analyses results.

|  | **ID** | **Description** | **GeneRatio** | **BgRatio** | **p-value** | **p-adjust** | **q-value** | **geneID** | **Count** |
| --- | --- | --- | --- | --- | --- | --- | --- | --- | --- |
| BP | GO:0006409 | tRNA export from nucleus | 3/45 | 34/18670 | 7.43E-05 | 0.021662 | 0.017095 | SSB/POM121/NUP205 | 3 |
| BP | GO:0071431 | tRNA-containing ribonucleoprotein complex export from nucleus | 3/45 | 34/18670 | 7.43E-05 | 0.021662 | 0.017095 | SSB/POM121/NUP205 | 3 |
| BP | GO:0051031 | tRNA transport | 3/45 | 36/18670 | 8.84E-05 | 0.021662 | 0.017095 | SSB/POM121/NUP205 | 3 |
| BP | GO:0097064 | ncRNA export from nucleus | 3/45 | 38/18670 | 0.000104 | 0.021662 | 0.017095 | SSB/POM121/NUP205 | 3 |
| BP | GO:0016052 | carbohydrate catabolic process | 5/45 | 199/18670 | 0.000113 | 0.021662 | 0.017095 | BPGM/POM121/G6PC/GM2A/NUP205 | 5 |
| BP | GO:0008202 | steroid metabolic process | 6/45 | 331/18670 | 0.000135 | 0.021662 | 0.017095 | OSBP/HSD17B6/RDH16/G6PC/AKR1C3/AKR1C2 | 6 |
| BP | GO:0006869 | lipid transport | 6/45 | 365/18670 | 0.000229 | 0.027848 | 0.021978 | OSBP/ABCA8/NMB/GM2A/ABCA5/ABCA6 | 6 |
| BP | GO:0030647 | aminoglycoside antibiotic metabolic process | 2/45 | 10/18670 | 0.000253 | 0.027848 | 0.021978 | AKR1C3/AKR1C2 | 2 |
| BP | GO:0034754 | cellular hormone metabolic process | 4/45 | 129/18670 | 0.00026 | 0.027848 | 0.021978 | HSD17B6/RDH16/AKR1C3/AKR1C2 | 4 |
| BP | GO:0010876 | lipid localization | 6/45 | 400/18670 | 0.000374 | 0.031906 | 0.02518 | OSBP/ABCA8/NMB/GM2A/ABCA5/ABCA6 | 6 |
| BP | GO:0006732 | coenzyme metabolic process | 6/45 | 403/18670 | 0.000389 | 0.031906 | 0.02518 | BPGM/POM121/GLYAT/QDPR/NUP205/DCAKD | 6 |
| BP | GO:0009108 | coenzyme biosynthetic process | 5/45 | 261/18670 | 0.000398 | 0.031906 | 0.02518 | BPGM/POM121/QDPR/NUP205/DCAKD | 5 |
| BP | GO:0042448 | progesterone metabolic process | 2/45 | 16/18670 | 0.000667 | 0.049423 | 0.039005 | AKR1C3/AKR1C2 | 2 |
| MF | GO:0016229 | steroid dehydrogenase activity | 4/47 | 35/17697 | 2.15E-06 | 0.000435 | 0.000331 | HSD17B6/RDH16/AKR1C3/AKR1C2 | 4 |
| MF | GO:0004745 | retinol dehydrogenase activity | 3/47 | 20/17697 | 1.94E-05 | 0.001657 | 0.001261 | HSD17B6/RDH16/AKR1C3 | 3 |
| MF | GO:0005319 | lipid transporter activity | 5/47 | 131/17697 | 2.46E-05 | 0.001657 | 0.001261 | OSBP/ABCA8/GM2A/ABCA5/ABCA6 | 5 |
| MF | GO:0033764 | steroid dehydrogenase activity, acting on the CH-OH group of donors, NAD or NADP as acceptor | 3/47 | 29/17697 | 6.11E-05 | 0.003086 | 0.002348 | HSD17B6/RDH16/AKR1C3 | 3 |
| MF | GO:0016616 | oxidoreductase activity, acting on the CH-OH group of donors, NAD or NADP as acceptor | 4/47 | 119/17697 | 0.000277 | 0.011202 | 0.008523 | HSD17B6/RDH16/AKR1C3/AKR1C2 | 4 |
| MF | GO:0016614 | oxidoreductase activity, acting on CH-OH group of donors | 4/47 | 128/17697 | 0.000366 | 0.012325 | 0.009377 | HSD17B6/RDH16/AKR1C3/AKR1C2 | 4 |
| MF | GO:0004032 | alditol:NADP+ 1-oxidoreductase activity | 2/47 | 13/17697 | 0.000529 | 0.015252 | 0.011604 | AKR1C3/AKR1C2 | 2 |
| MF | GO:0008106 | alcohol dehydrogenase (NADP+) activity | 2/47 | 21/17697 | 0.001404 | 0.035447 | 0.026969 | AKR1C3/AKR1C2 | 2 |
| MF | GO:0004033 | aldo-keto reductase (NADP) activity | 2/47 | 26/17697 | 0.002154 | 0.043518 | 0.033109 | AKR1C3/AKR1C2 | 2 |
| MF | GO:0016628 | oxidoreductase activity, acting on the CH-CH group of donors, NAD or NADP as acceptor | 2/47 | 26/17697 | 0.002154 | 0.043518 | 0.033109 | AKR1C3/AKR1C2 | 2 |
| MF | GO:0017056 | structural constituent of nuclear pore | 2/47 | 28/17697 | 0.002497 | 0.045858 | 0.034889 | POM121/NUP205 | 2 |
| MF | GO:0016410 | N-acyltransferase activity | 3/47 | 113/17697 | 0.003351 | 0.047407 | 0.036068 | USP22/NMT1/GLYAT | 3 |
| MF | GO:0009055 | electron transfer activity | 3/47 | 114/17697 | 0.003435 | 0.047407 | 0.036068 | HSD17B6/RDH16/QDPR | 3 |
| MF | GO:0042626 | ATPase activity, coupled to transmembrane movement of substances | 3/47 | 115/17697 | 0.00352 | 0.047407 | 0.036068 | ABCA8/ABCA5/ABCA6 | 3 |
| MF | GO:0043492 | ATPase activity, coupled to movement of substances | 3/47 | 115/17697 | 0.00352 | 0.047407 | 0.036068 | ABCA8/ABCA5/ABCA6 | 3 |
| KEGG | hsa02010 | ABC transporters | 3/30 | 45/8094 | 0.000587 | 0.032876 | 0.030898 | 10351/23461/23460 | 3 |
| KEGG | hsa00140 | Steroid hormone biosynthesis | 3/30 | 61/8094 | 0.001431 | 0.040059 | 0.037649 | 8630/8644/1646 | 3 |


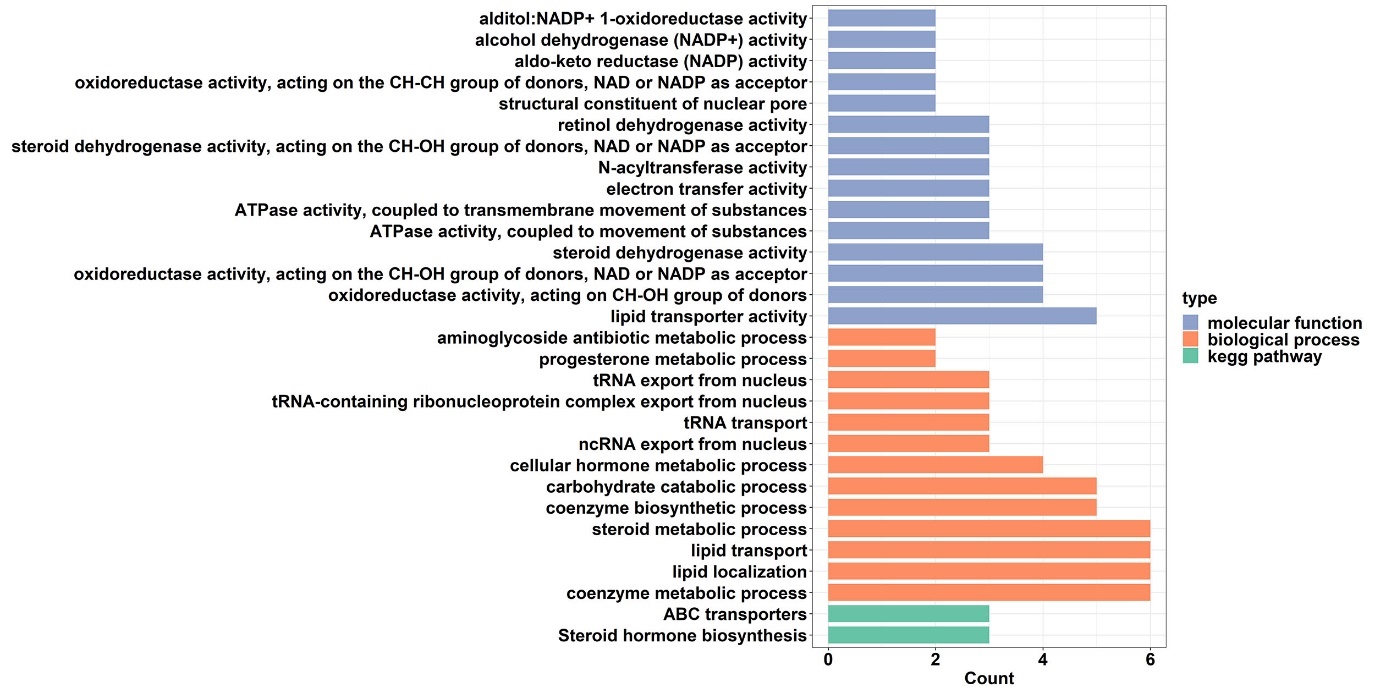


**Figure S5.** GO terms and KEGG pathways enriched by survival-related eRGs.


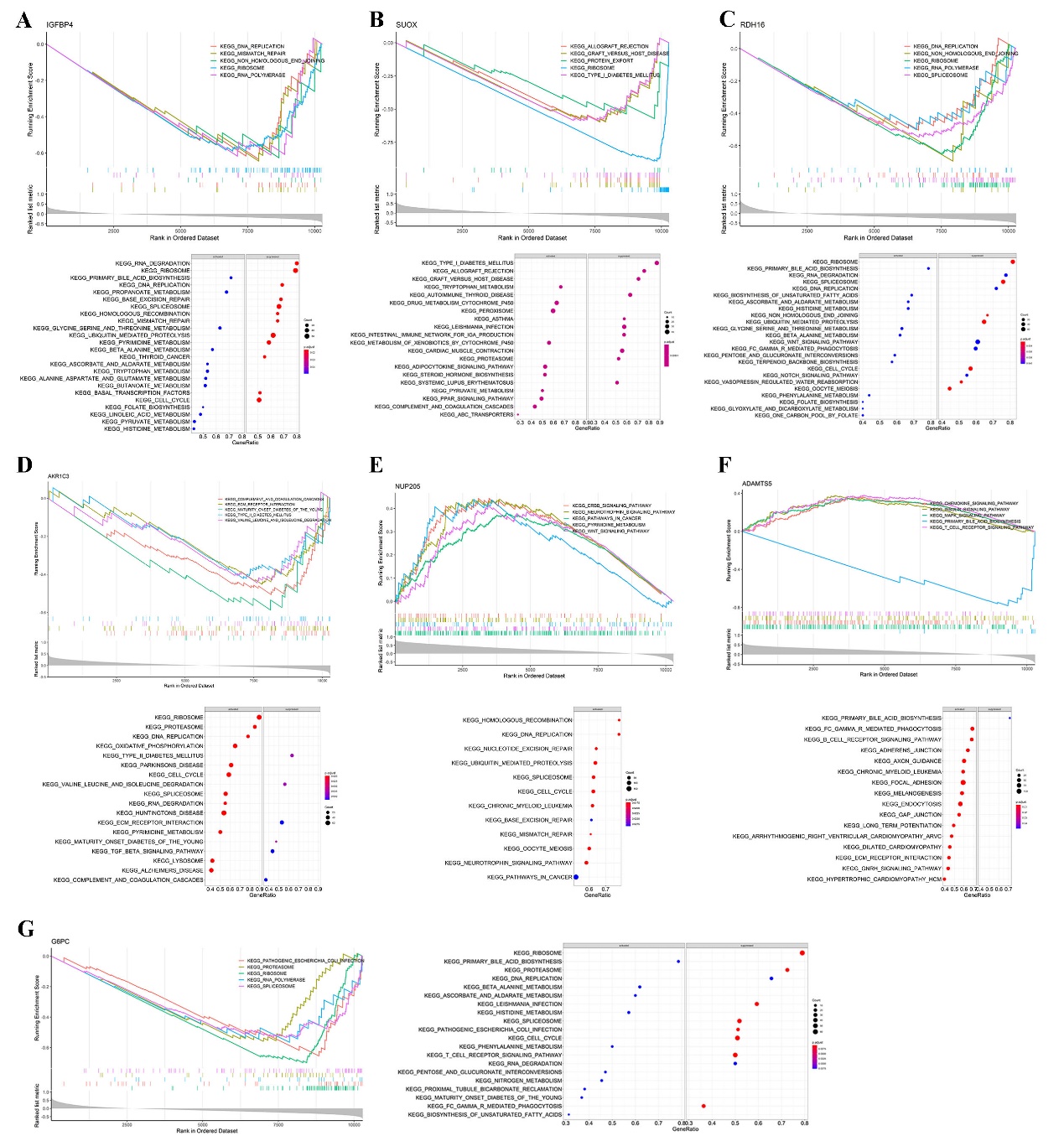


**Supplementary Figure S6.** Single gene GSEA results (IGFBP4, SUOX, RDH16, G6PC, AKRIC3, NUP205, ADAMTS5).

**Supplementary Table S4.** GSVA results: different pathways between high risk group and low risk group.

| **pathway** | **logFC** | **average expression** | **t** | **p-value** | **adjusted p-value** |
| --- | --- | --- | --- | --- | --- |
| PATHOGENIC_ESCHERICHIA_COLI_INFECTION | -0.32145 | -0.02732 | -14.1546 | 7.60E-36 | 1.38E-33 |
| PEROXISOME | 0.368841 | 0.015424 | 12.35001 | 4.81E-29 | 4.38E-27 |
| TYROSINE_METABOLISM | 0.326634 | 0.037261 | 12.24988 | 1.12E-28 | 6.82E-27 |
| LINOLEIC_ACID_METABOLISM | 0.298672 | 0.039943 | 11.61357 | 2.33E-26 | 1.06E-24 |
| CELL_CYCLE | -0.29421 | -0.04582 | -11.4861 | 6.69E-26 | 2.44E-24 |
| PRIMARY_BILE_ACID_BIOSYNTHESIS | 0.469573 | 0.020334 | 11.31578 | 2.72E-25 | 8.25E-24 |
| GLYCINE_SERINE_AND_THREONINE_METABOLISM | 0.37322 | 0.012668 | 11.08396 | 1.81E-24 | 4.70E-23 |
| TRYPTOPHAN_METABOLISM | 0.371514 | 0.013876 | 10.91132 | 7.32E-24 | 1.51E-22 |
| DRUG_METABOLISM_CYTOCHROME_P450 | 0.316483 | 0.023303 | 10.67479 | 4.90E-23 | 8.91E-22 |
| FATTY_ACID_METABOLISM | 0.398987 | 0.015682 | 10.61947 | 7.62E-23 | 1.26E-21 |
| RETINOL_METABOLISM | 0.345374 | 0.031385 | 10.56986 | 1.13E-22 | 1.72E-21 |
| HISTIDINE_METABOLISM | 0.312847 | 0.01399 | 10.55397 | 1.28E-22 | 1.80E-21 |
| PROPANOATE_METABOLISM | 0.391719 | 0.008408 | 10.27738 | 1.14E-21 | 1.48E-20 |
| VALINE_LEUCINE_AND_ISOLEUCINE_DEGRADATION | 0.400368 | 0.008734 | 10.24083 | 1.52E-21 | 1.84E-20 |
| BUTANOATE_METABOLISM | 0.331126 | 0.019154 | 10.08877 | 4.98E-21 | 5.34E-20 |
| BETA_ALANINE_METABOLISM | 0.336494 | 0.018207 | 10.08861 | 4.99E-21 | 5.34E-20 |
| PPAR_SIGNALING_PATHWAY | 0.264648 | 0.025676 | 9.913904 | 1.93E-20 | 1.95E-19 |
| FOLATE_BIOSYNTHESIS | 0.313874 | 0.035034 | 9.682517 | 1.14E-19 | 1.09E-18 |
| STEROID_HORMONE_BIOSYNTHESIS | 0.272146 | 0.042578 | 9.414644 | 8.59E-19 | 7.82E-18 |
| PHENYLALANINE_METABOLISM | 0.287652 | 0.013967 | 9.128004 | 7.23E-18 | 5.98E-17 |
| SPLICEOSOME | -0.24423 | -0.04622 | -8.50799 | 6.36E-16 | 4.82E-15 |
| PYRUVATE_METABOLISM | 0.257003 | 0.006685 | 8.497826 | 6.83E-16 | 4.97E-15 |
| METABOLISM_OF_XENOBIOTICS_BY_CYTOCHROME_P450 | 0.257377 | 0.022191 | 8.468047 | 8.42E-16 | 5.90E-15 |
| COMPLEMENT_AND_COAGULATION_CASCADES | 0.288041 | 0.006364 | 8.384375 | 1.52E-15 | 1.02E-14 |
| ARGININE_AND_PROLINE_METABOLISM | 0.225755 | 0.005664 | 8.159917 | 7.21E-15 | 4.68E-14 |
| ALANINE_ASPARTATE_AND_GLUTAMATE_METABOLISM | 0.215581 | 0.011193 | 7.818216 | 7.34E-14 | 4.31E-13 |
| GLYOXYLATE_AND_DICARBOXYLATE_METABOLISM | 0.264207 | -0.00596 | 7.627349 | 2.61E-13 | 1.48E-12 |
| DNA_REPLICATION | -0.2957 | -0.03874 | -6.86344 | 3.36E-11 | 1.53E-10 |
| MISMATCH_REPAIR | -0.2222 | -0.04102 | -6.13147 | 2.50E-09 | 8.45E-09 |
| RIBOSOME | -0.23744 | -0.03131 | -4.57966 | 6.62E-06 | 1.43E-05 |

**Supplementary Table S5.** Transcription factors related to the genes in prognostic signature.

| **TF** | **immune Gene** | **correlation** | **p-value** | **Regulation** | **TF** | **immune Gene** | **correlation** | **p-value** | **Regulation** |
| --- | --- | --- | --- | --- | --- | --- | --- | --- | --- |
| STAT1 | SSRP1 | 0.411036 | 1.22E-14 | positive | E2F1 | SSRP1 | 0.544527 | 2.08E-26 | positive |
| HIF1A | SSRP1 | 0.503254 | 3.33E-22 | positive | E2F1 | NUP205 | 0.483841 | 2.04E-20 | positive |
| HIF1A | SSB | 0.457408 | 3.71E-18 | positive | SOX9 | SSB | 0.434847 | 2.23E-16 | positive |
| HIF1A | NUP205 | 0.629829 | 3.27E-37 | positive | LMNB1 | SSRP1 | 0.726079 | 2.57E-54 | positive |
| HIF1A | ADAMTS5 | 0.577369 | 3.39E-30 | positive | LMNB1 | SSB | 0.54765 | 9.44E-27 | positive |
| SOX4 | SSRP1 | 0.472826 | 1.89E-19 | positive | LMNB1 | NUP205 | 0.719215 | 7.34E-53 | positive |
| SOX4 | SSB | 0.519117 | 9.41E-24 | positive | ARID3A | SSB | 0.466261 | 6.83E-19 | positive |
| SOX4 | RDH16 | -0.4404 | 8.36E-17 | negative | ARID3A | RDH16 | -0.48363 | 2.13E-20 | negative |
| SOX4 | G6PC | -0.45111 | 1.20E-17 | negative | TAT | SSRP1 | -0.40794 | 2.01E-14 | negative |
| SOX4 | NUP205 | 0.437442 | 1.41E-16 | positive | TAT | SSB | -0.50627 | 1.71E-22 | negative |
| SOX4 | ADAMTS5 | 0.450581 | 1.32E-17 | positive | TAT | SUOX | 0.429353 | 5.78E-16 | positive |
| MYBL2 | SSRP1 | 0.604811 | 1.05E-33 | positive | TAT | RDH16 | 0.56738 | 5.34E-29 | positive |
| MYBL2 | SSB | 0.520213 | 7.30E-24 | positive | TAT | G6PC | 0.587245 | 2.03E-31 | positive |
| MYBL2 | RDH16 | -0.40997 | 1.45E-14 | negative | AR | SUOX | 0.450663 | 1.30E-17 | positive |
| MYBL2 | G6PC | -0.45294 | 8.56E-18 | negative | AR | RDH16 | 0.585376 | 3.48E-31 | positive |
| MYBL2 | NUP205 | 0.579281 | 1.98E-30 | positive | SRC | SSRP1 | 0.416378 | 5.12E-15 | positive |
| MYBL2 | RRAGD | 0.418335 | 3.71E-15 | positive | SRC | SSB | 0.562746 | 1.86E-28 | positive |
| MYC | SSB | 0.435219 | 2.09E-16 | positive | SRC | RDH16 | -0.45714 | 3.90E-18 | negative |
| FOXM1 | SSRP1 | 0.731993 | 1.32E-55 | positive | EHF | SSRP1 | 0.417919 | 3.97E-15 | positive |
| FOXM1 | SSB | 0.515326 | 2.24E-23 | positive | EHF | NUP205 | 0.44665 | 2.72E-17 | positive |
| FOXM1 | NUP205 | 0.774878 | 4.10E-66 | positive |  |  |  |  |  |
